# Cytotoxic and Anti-Plasmodial Activities of *Stephania dielsiana* Y.C. Wu Extracts and the Isolated Compounds

**DOI:** 10.3390/molecules25163755

**Published:** 2020-08-18

**Authors:** James Knockleby, Bruno Pradines, Mathieu Gendrot, Joel Mosnier, Thanh Tam Nguyen, Thi Thuy Trinh, Hoyun Lee, Phuong Mai Le

**Affiliations:** 1 Health Science North Research Institute, 56 Walford Road, Sudbury, ON P3E 2H2, Canada; jknockleby@hsnri.ca; 2 Department of Chemistry and Biochemistry, Laurentian University, 935 Ramsey Lake Road, Sudbury, ON P3E 2C6, Canada; 3 Département Microbiologie et Maladies Infectieuses, Institut de Recherche Biomédicale des Armées, 13005 Marseille, France; bruno.pradines@gmail.com (B.P.); ma.gendrot@laposte.net (M.G.); joelmosnier@orange.fr (J.M.); 4 Aix Marseille University, IRD, SSA, AP-HM, VITROME, 13005 Marseille, France; 5 IHU Méditerranée Infection, 13005 Marseille, France; 6 Centre National de Référence du Paludisme, Institut de Recherche Biomédicale des Armées, 13005 Marseille, France; 7 Graduate University of Science and Technology and Institute of Chemistry, Vietnam Academy of Science and Technology, 18 Hoang Quoc Viet Road, Hanoi 70750, Vietnam; nttam76@yahoo.com (T.T.N.); thuy@ich.vast.vn (T.T.T.); 8 Departments of Medicine, University of Ottawa Medical School, Ottawa, ON K1H 5M8, Canada; 9 National Research Council Canada, 100 Sussex Drive, Ottawa, ON K1A 0R6, Canada

**Keywords:** *Stephania dielsiana* Y.C. Wu, aporphine alkaloids, Aurora kinase, cancer cells, malaria, *Plasmodium falciparum*

## Abstract

Natural products remain a viable source of novel therapeutics, and as detection and extraction techniques improve, we can identify more molecules from a broader set of plant tissues. The aim of this study was an investigation of the cytotoxic and anti-plasmodial activities of the methanol extract from *Stephania dielsiana* Y.C. Wu leaves and its isolated compounds. Our study led to the isolation of seven alkaloids, among which oxostephanine (**1**) is the most active against several cancer cell lines including HeLa, MDA-MB231, MDA-MB-468, MCF-7, and non-cancer cell lines, such as 184B5 and MCF10A, with IC_50_ values ranging from 1.66 to 4.35 μM. Morever, oxostephanine (**1**) is on average two-fold more active against cancer cells than stephanine (**3**), having a similar chemical structure. Cells treated with oxostephanine (**1**) are arrested at G2/M cell cycle, followed by the formation of aneuploidy and apoptotic cell death. The G2/M arrest appears to be due, at least in part, to the inactivation of Aurora kinases, which is implicated in the onset and progression of many forms of human cancer. An in-silico molecular modeling study suggests that oxostephanine (**1**) binds to the ATP binding pocket of Aurora kinases to inactivate their activities. Unlike oxostephanine (**1**), thailandine (**2**) is highly effective against only the triple-negative MDA-MB-468 breast cancer cells. However, it showed excellent selectivity against the cancer cell line when compared to its effects on non-cancer cells. Furthermore, thailandine (**2**) showed excellent anti-plasmodial activity against both chloroquine-susceptible 3D7 and chloroquine-resistant W2 *Plasmodium falciparum* strains. The structure–activity relationship of isolated compound was also discussed in this study. The results of this study support the traditional use of *Stephania dielsiana* Y.C. Wu and the lead molecules identified can be further optimized for the development of highly effective and safe anti-cancer and anti-plasmodial drugs.

## 1. Introduction

Aurora kinases are an important class of cell cycle kinases that regulate and coordinate many aspects of mitotic cell division. The overexpression of Aurora kinases has been correlated with cancer development, and their inhibitors can effectively suppress cancer cell growth both in vitro and in vivo. In humans, three Aurora kinases are present in the genome (Aurora A, B, and C) [1,2]. Aurora A localizes to the microtubules and centromeres and plays an important role in centrosome function and bipolar spindle assembly [1,2]. Aurora B is required for proper kinetochore microtubule attachment and chromosome segregation during anaphase and the successful completion of cytokinesis [1,2,3]. Aurora C has specific roles in meiosis that partially overlap with Aurora B functions [4]. However, there is also evidence that Aurora C can substitute for Aurora B in cancer cells [3]. Much effort has been made over the last decade and a half to find inhibitors that can specifically target Aurora kinases as they may be effective anticancer therapeutic targets [1,2]. Additionally, three Aurora-related kinases have been reported in *Plasmodium falciparum*. One of these, Pfark-1, an Aurora kinase A, is required at the S to M phase transition of the parasite cycle and is highly conserved in human and rodent malaria species and other apicomplexan parasites [5,6]. PfArk-3 is also conserved in apicomplexan parasites while PfArk-2 is specific to *Plasmodium* [7]. Hesparadin, a human Aurora B inhibitor, and analogs were identified to be highly potent against *P. falciparum* [8].

Malaria remains one of the most prevalent infectious diseases in the world. According to the World Health Organization (WHO), approximately 219 million cases of infection and 405,000 deaths occur every year by this disease [9]. Although malarial disease is quite effectively treated with existing drugs, the emergence of drug-resistant strains is still a serious problem [10,11]. Malaria therapy has a strong historical link to natural compounds. The most successful antimalarial drugs have natural origins. Quinine was isolated from the bark of the South American *Cinchona* tree, which was historically used to treat fever [12]. Chloroquine, one of most used antimalarial drugs three decades ago and disused now due to of the development of drug resistance by parasites, is a synthetic compound derived from quinine. Moreover, artemisinin and its derivatives, which are the drugs currently recommended in combination (artemisinin-based combination therapy) by the WHO to treat malaria, were isolated from *Artemisinia annua*, a plant historically used to treat fever [13]. More than 1500 compounds isolated from plants were evaluated in vitro against *P. falciparum* parasites [14].

Natural products have been discovered as rich and powerful sources for anticancer and antimalarial therapies, including vinblastine from *Catharanthus roseus* [15,16], paclitaxel or taxol from *Taxus brevifolia* [17,18], curcurmin from *Curcuma longa* [19] and artemisinin from *Artemisia annua* [13,20,21]. Moreover, synthetic compounds were identified to be effective on both cancer cells and *Plasmodium*, such as gold- or iron-metallocenes [22,23,24]. One of these, ferroquine, an analog of chloroquine, showed high in vitro activity against *P. falciparum* [25,26,27] and antitumor activity [28]. The systematic investigation and validation of traditional and ethno medicines could thus lead to the development of new effective and safe anticancer and antimalarial drugs.

The genus *Stephania* belongs to the family Menisermaceae containing approximately 60 species, widely distributed in Africa, India, South-East Asia, and the northern and eastern parts of Australia. *Stephania* plants have long been used in traditional medicine for the treatment of cancer, skin diseases, diabetes, anemia, headache and insomnia [29,30]. The main chemical constituents of *Stephania* species are isoquinoline and aporphine alkaloids [29]. Many aporphinoids isolated from *Stephania* plants, such as oxostephanine, thailandine, and stephanine, have been shown to possess anti-growth and anti-malarial activities [31,32,33,34,35,36]. Interestingly, thailandine has positively charged quaternary nitrogen that has been attributed to its reduced toxicity to normal cells [31]. Stephanine isolate showed a unique characteristic phenotype such as a transient G2/M arrest and chromosome segregation failure that may eventually lead to aneuploidy [34]. Although the aporphine alkaloids isolated from *Stephania dielsiana* Y.C. Wu [37] and the cytotoxicity of some of these alkaloids were previously reported [38], their mechanism of action for the antiproliferative and antimalarial activity has not been elucidated.

In this study, we systematically evaluated the anti-proliferative and anti-plasmodial effects of the crude methanol extracts of *Stephania dielsiana* Y.C. Wu (*S. dielsiana*) leaves, its fractions and isolated compounds on the human cancer and non-cancer cells as well as chloroquine-susceptible 3D7 and chloroquine-resistant W2 *P. falciparum* parasites. A bioassay-guided fractionation led to the isolation of seven alkaloids, among which oxostephanine (**1**) and thailandine (**2**) show strong anticancer and/or anti-plasmodial activities. The mechanism of action of oxostephanine (**1**) on human cancer cell line HeLa was investigated. This is the first time that the functional analysis of oxostephanine identified Aurora kinase as a putative cellular target, and we determined that oxostephanine (**1**) interacts in the ATP binding pockets of Aurora A and B kinases. Moreover, the anti-plasmodial activity of isolated alkaloids was also evaluated in this study. Our data from an in vitro study showed that thailandine (**2**) possess excellent anti-plasmodial activity against both chloroquine-susceptible 3D7 and chloroquine-resistant W2 *P. falciparum* strains. To our knowledge, this is the first report that oxostephanine, thailandine and other alkaloid compounds from Vietnamese *S. dielsiana* Y.C. Wu possess both anticancer and anti-plasmodial activities.

## 2. Results and Discussion

### 2.1. Bioassay-Guided Fractionation and Isolation of S. dielsiana Y.C. Wu

The methanol crude (MB2L) extract showed good activity against HeLa, MDA-MB-231, MDA-MB-468, and MCF7 cancer cells lines (Table 1). Further fractionation of the methanol extract showed that the dichloromethane (MB2L-CH) and butanol (MB2L-B) fractions possess high activity against cancer cells with IC_50_ values in the range of 0.57 to 14.35 μg/mL, while the hexane (MB2L-H) fraction was not active (Table 1). In particular, MB2L-CH fraction was effective on all of the cell lines examined, although it did not show any selectivity between four cancer cell lines and two non-cancer cell lines (184B5 and MCF10A). The MB2L-B fraction was effective against only MDA-MB-468; however, it is very selective against the cancer cell line when compared with its cytotoxicity against 184B5 (3.2-fold) and MCF10A (7.8-fold) non-cancer cells (Table 1). In addition to cancer cells, the MB2L-CH fraction was also quite potent against both chloroquine-susceptible 3D7 (IC_50_, 4.5 μg/mL) and chloroquine-resistant W2 (IC_50_, 5.8 μg/mL) *P. falciparum* strains (Table 1).

**Figure 1:**
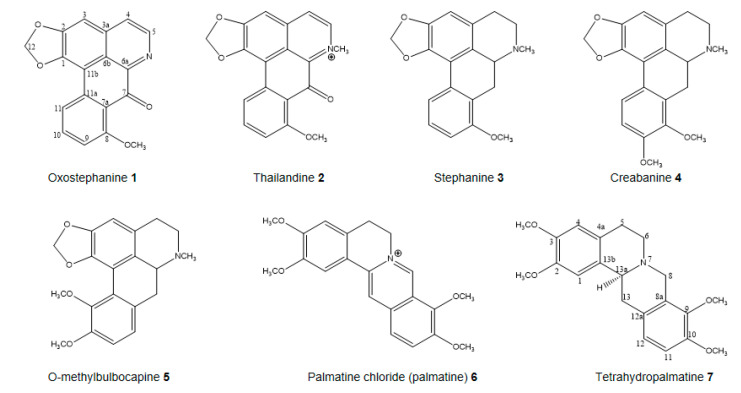
Compounds isolated from *Stephania dielsiana* Y.C. Wu.

The two most effective MB2L-CH and MB2L-B fractions (Table 1) were further fractionated and purified by repeated chromatography on silica gel columns, which led to the isolation of the seven pure compounds: compounds **1**, **2**, and **6** and compounds **3**, **4**, **5**, and **7** were isolated from the MB2L-B and MB2L-CH fractions, successively. The chemical structures of these seven alkaloids, as oxostephanine (**1**), thailandine (**2**), stephanine (**3**), crebanine (**4**), O-methylbulbocapnine (**5**), palmatine (**6**), and tetrahydropalmatine (**7**), were confirmed by the analysis of NMR and HR-ESIMS spectrum as well as by comparing spectroscopic data with those reported previously (Figure 1, Supplementary Tables S1 and S2) [34,38].

### 2.2. In Vitro Antiproliferative and Structure-Activity Relationship (SAR) of Isolated Compounds

All seven isolated pure compounds were evaluated for their anti-proliferative activity against four cancer (HeLa, MDA-MB231, MDA-MB-468 and MCF7) and two non-cancer (184B5 and MCF10A) cell lines (Table 2A). The selectivity index (SI) of cancer versus non-cancer cell lines was calculated (Table 2B) for each of the pure compounds with a calculated IC_50_. The selectivity of most of the compounds, as well as the control compounds chloroquine and paclitaxel is in the range of 1–2, with some selectivity seen in MCF7 and MDA-MB-468 in comparison with MCF10A (Table 2B).

**Figure 2:**
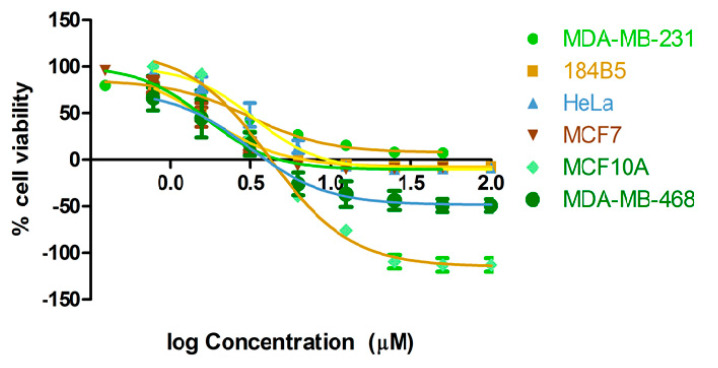
Anti-proliferative activity of oxostephanine (**1**) against cancer (MDA-MB-231, HeLa, MCF7 and MDA-MB-468) and non-cancer (184B5 and MCF10A) cells. Dose response curves are obtained from 72 h treatment and staining with SRB.

A study by structure–bioactivity relationship on these isolates showed that aporphine alkaloids (1–5) showed anti-proliferative activities against cancer and non-cancer cell lines in the range of 0.7–74 μM, whereas protoberberine (6) and tetrahydroprotoberberine (7) did not show any effect (Figure 1, Table 2A). This finding suggests that the activity of the butanol and dichloromethane fractions can be attributed to the aporphine alkaloids present in each fraction, which is consistent with previous reports on the cytotoxicity on this type of alkaloids isolated from *Stephania verosa* [31,34]. Of five aporphine alkaloids (1–5), oxostephanine (1) is the most active against all of the cell lines examined with IC_50_ values ranging from 1.66 to 4.35 μM (Figure 2, Table 2A), although thailandine (2) and stephanine (3) also show strong activities (Table 2A). These three active aporphine alkaloids have similar structures (Figure 1). Oxostephanine (1) maintains most of the stephanine (3) structure with the addition of a 7-oxo group and a double bond between carbon 6a and nitrogen (Figure 1). Comparing the two compounds, it is clear that these changes increase the effectiveness of oxostephanine (1) by 2-fold (HeLa, 3.33 μM vs. 1.73 μM) to almost 4-fold (184B5, 6.25 μM vs. 1.66 μM) against the cell lines. However, these changes result in less selectivity against cancer cells when compared to its cytotoxicity on non-cancer 184B5 and MCF10A cells (Table 2A,B). An addition of a methyl group linked to the nitrogen atom of oxostephanie (1) (which forms a quaternary nitrogen atom and subsequent addition of a positive charge) shown in the thailandine (2) reduces the effectiveness of the molecule against most cancer and non-cancer cells, which corroborates with previous report by Makarasen [31]. However, thalandine (2) is very effective against MDA-MB-468 with the IC_50_ of 0.78 μM, where it is 3.9-fold (0.78 μM vs. 3.02 μM) and 6.4-fold (0.78 μM vs. 5.01 μM) more selective over 184B5 and MCF10A non-cancer cells, respectively (Table 2A). This raises the possibility that the selectivity shown by MB2L-B (Table 1) may be the effect of thailandine (2). The effectiveness of stephanine (3) against certain cancer cell types may be substantially better with the addition of a 7-oxo group or the modification of nitrogen without positive charge at the position of carbon-6 to a quaternary nitrogen atom (forming a positive charge). Thailandine (2) is electron-deficient, and the positive charge of the molecule may make it more effective against a certain sub-types of cancer cells, potentially in a chloroquine-like weak base manner [39]. According to Boyd [40], a compound can be considered as active if its IC_50_ is less than 100 μM. In that sense, crebanine (4) and O-methylbulbocapnine (5) are also considered weakly active because their IC_50_ values are in the ranges of 17–48 μM and 39–73 μM, respectively (Table 2A), consistently with previous results [34]. The antiproliferative activities of oxostephanine (1), thailandine (2) and crebanine (4) against several cancer cell lines have been reported by Makarasen [31]; however, the MCF-10A and 184B5 non-cancer cell lines were not included in their study. The antiproliferative of the alkaloids isolated from *S. dielsiana* Y.C. Wu was investigated for the first time on two human cancer cell-lines of BT174 (breast) and HCT116 (colon) [38]. They mentioned thailadine (2), stephanine (3) and crebanine (4) are active on BT474 cell lines. However, the structure-activity relationship of these isolated compounds was not discussed [38].

### 2.3. Oxostephanine Leads to A Transient G2/M Arrest and Apoptotic Cell Death

**Figure 3:**
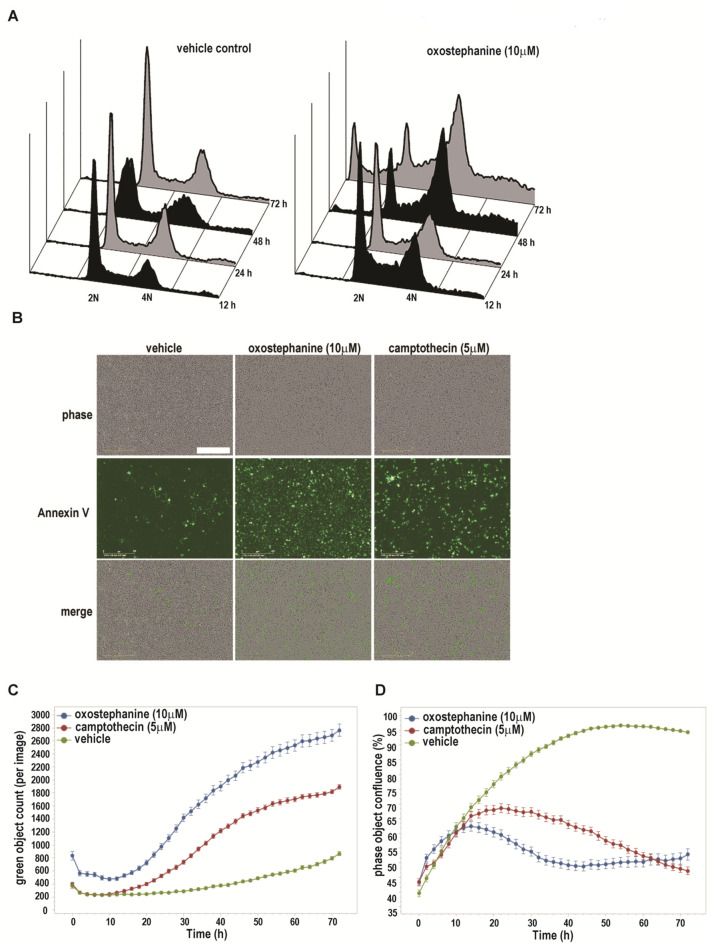
Oxostephanine (**1**) causes G2/M arrest and apoptosis. (**A**) Asynchronous HeLa cells were treated with 10 μM oxostephanine 1 or sham control for 12–72 h, and analysed their cell cycle distributions by flow cytometry. (**B**,**C**) MDA-MB-231 cells treated with oxostephanine undergo apoptosis by 48 h. Representative images of cells at 48 h post-oxostephanine are shown. Bar = 100 μm. Quantification of Annexin V positive cells over the 72 h time course are shown in (**C**). Error bars = SEM, *n* = 4. (**D**) Quantification of cell population indicates that oxostephanine inhibits proliferation of MDA-MB-231 cells within 12 h of treatment. Error bars = SEM, *n* = 4.

Since oxostephanine (**1**) was the most active compound against all of the cancer cell lines examined, its mode of action was investigated. The effects of oxostephanine on a population level was examined using flow cytometry. An initial study of cell cycle distribution showed that the majority of cells (HeLa) were arrested at G2/M or accumulated as multiploidy after 48 h post-oxostephanine (**1**) treatment (Figure 3A). By 72 h post-treatment, a substantial portion of the cell population contained sub-G1 DNAs with profile that is typical for apoptotic cells. These data suggest that oxostephanine (**1**) induces a transient G2/M arrest and apoptotic cell death, reminiscent of previous results for the structurally similar stephanine (**3**) [34].

To confirm the mode of cell death, we carried out an Annexin V staining assay with live cells. As shown in Figure 3B–D, MDA-MB-231 cells treated with oxostephanine started to undergo apoptosis within 16 h post-treatment. The number of apoptotic cells rapidly increased until 48 h, from which time it remained relatively static until 72 h post-treatment. Compared to camptothecin, a well-known apoptosis inducer (as a positive control in this study), oxostephanine (**1**) showed a much stronger apoptotic effect at 10 μM (Figure 3C, 1-way ANOVA, *p* < 0.05). This finding supports the previous study [41], which showed oxostephanine (**1**) possess a selective toxicity in HeLa cells and a marked reduction in the mitotic index for tumoral cells. However, the mechanism of action of this compound was not investigated in the above study.

### 2.4. Oxostephanine Inhibits Aurora Kinase Activity

**Figure 4:**
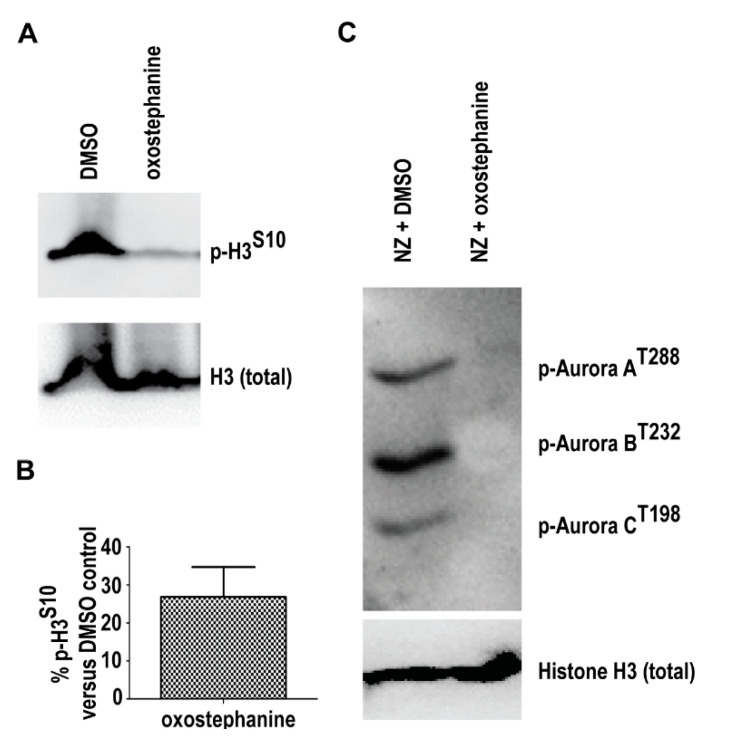
Oxostephanine (**1**) inhibits phosphorylation of histone H3 and Aurora kinases A, B and C. (**A**) Histone H3 phosphorylation on Ser-10 (p-H3^S10^) is substantially downregulated in response to oxostephanine **1** for 12 h. (**B**) Quantification of histone H3^S10^ phosphorylation (p-H3^S10^). P-H3^S10^ was normalized to total histone H3 protein (H3). (**C**) The autophosphorylation of Aurora kinases A, B and C is inhibited in the cells treated with 20 μM oxostephanine for 12 h. Total histone H3 protein was used as a control.

Inactivation of Aurora kinases A and B by specific inhibitors leads to the induction of spindle assembly checkpoint (SAC), arresting cells at mitosis. However, the SAC checkpoint is not permanent, as it is soon overridden and cells exit mitosis without proper chromosome segregation [42]. The inhibition of Aurora A or Aurora B may eventually induce apoptosis or DNA reduplication without undergoing cytokinesis [42]. Data shown in Figure 3 are reminiscent of the previous observations in human cells with ablated Aurora kinases. Therefore, we examined whether Aurora kinases are the targets of oxostephanine (**1**) using Western blot analysis. As shown in Figure 4A,B, HeLa cells treated with oxostephanine (**1**) for 12 h dramatically downregulated the phosphorylation of histone H3 on Ser-10, a known molecular target of Aurora kinases [43]. The effect of oxostephanine on autophosphorylation of all three Aurora kinases in HeLa cells arrested in mitosis was also investigated (Figure 4C). Data from our Western blot also showed that upon treatment of cells, Aurora A, B, and C phosphorylation was reduced, showing that oxostephanine (**1**) inhibits the auto-phosphorylation of all three Aurora kinases (Figure 4C). These data indicate that Aurora kinases are indeed a target of oxostephanine.

### 2.5. Oxostephanine Inhibits Aurora Kinase Activity In Vitro and Is Predicted to Bind to the ATP Binding Pocket of Both Aurora A and B

**Figure 5:**
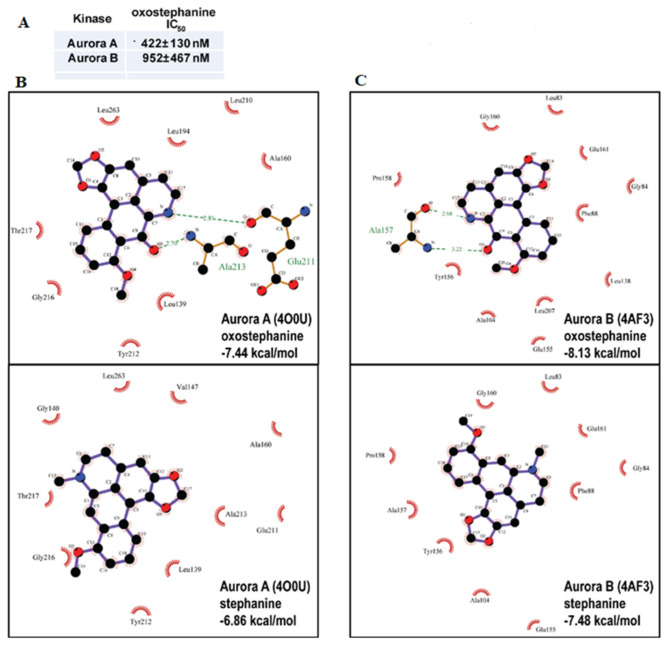
Oxostephanine (**1**) inhibits Aurora kinase activity, and is predicted to target the ATP binding pockets of Aurora kinases. (**A**) The IC_50_ values of oxostephanine determined by an in vitro kinase assay are shown. Value is +/− standard deviation, *n* = 2. (**B**) Oxostephanine is predicted to bind to the ATP binding pocket of Aurora kinases A. Two dimensional representations generated by LigPlot+ are shown of the best binding energy poses of oxostephanine based on Autodock results. One-letter and three-letter codes are elements and amino acids, respectively. Green dashed lines denote H-bonds, and number is the distance in angstrom. Red half circles denote hydrophobic interactions. Predicted binding energy is indicated in blue. Aurora A is based on crystal structure 4O0U. (**C**) Oxostephanine is predicted to bind to the ATP binding pocket of Aurora kinase B. The representation is the same as above and Aurora B is based on crystal structure 4AF3.

In order to further characterize the activity of oxostephanine (**1**) and determine if there is activity of oxostephanine (**1**) against aurora kinases, we carried out an in vitro kinase assay against only Aurora A and B kinase to determine if oxostephanine (**1**) can inhibit either of the kinases. As expected, data from an in vitro kinase assay showed that one half of the kinase activities were inhibited (IC_50_) by oxostephanine (**1**) at 422 ± 130 nM to 952 ± 467 nM for Aurora kinases A and B, respectively (Figure 5A). Employing in silico modeling, we found that oxostephanine would interact with the ATP binding pocket of Aurora A (crystal structure 4O0U) when docked blindly to the entire structure of Aurora A (Figure 5B-top). Oxostephanine would ‘lock’ in the hydrophobic pocket, where it would interact with the fluorophenyl Leu-210 residue. As well, oxostephanine is also likely to form hydrogen bonds with Glu-211 and Ala-213, the two amino acids that interact with the adenine of ATP in the pocket. This suggests that oxostephanine would occupy much of the same space as one ATP molecule and, thus, effectively compete with it for binding to the pocket. Similarly, oxostephanine also interacts with the hydrophobic ATP binding pocket of Aurora kinase B (4AF3) (Figure 5C-top).

Because of their anti-proliferative activity and structural similarity to oxostephanine (**1**), we also examined how stephanine (**3**) binds to Aurora A kinase. As shown in Figure 5B, they both bind favorably to the ATP binding site, although not as favorably as oxostephanine (**1**). This is likely due to the inability of stephanine (**3**) to form a hydrogen bond with any residues in the pocket, and in particular the Glu-211 and Ala-213 ATP interacting residues (Figure 5B-bottom). Stephanine (**3**) does interact with the ATP pocket through hydrophobic interactions, and notably with the Leu-210 in the fluorophenyl pocket. When blind docking was carried out to the human Aurora B kinase (4AF3), we found that the two aporphine alkaloids interact with the hydrophobic ATP binding pocket (Figure 5C). Oxostephanine’s binding was more energetically favorable than stephanine. In part, the carbonyl group and aromatic nitrogen can form a hydrogen bond with Ala-157 (Figure 5C-top). However, as with the Aurora A ATP pocket, stephanine (**3**) does not form a hydrogen bond (Figure 5C-bottom). The predicted binding of oxostephanine in the ATP pockets of both Aurora A and B, and by extension Aurora C (since it shares much similarity with Aurora B, but does not have a published crystal structure that we are aware of), could be a reason why it has higher efficacy against cancer cells than stephanine. Based on the cell cycle phenotypes, in vitro kinase assay and the in-silico modeling, it appears that the two aporphine alkaloids extracted from *S. dielsiana* Y.C. Wu are active in large part because they are Aurora kinase inhibitors.

Although oxostephanine (**1**) is more effective than stephanine (**3**), it also is cytotoxic towards proliferating non-cancer models MCF10A and 184B5 at similar concentrations, suggesting that oxostephanine may have off-target cytotoxic effects or may have a narrow therapeutic window. Alternatively, MCF10A and 184B5 are still proliferating cells, even though they are not tumorigenic, and thus would require Aurora kinases for proper cell division. Thus, the low therapeutic index observed may reflect the inhibition of Aurora kinase function in those cell lines as well. It does highlight the challenge within the Aurora kinase inhibitor development field. Since no Aurora kinase inhibitor has made it past Phase III clinical trials as of now, there may be limitations in targeting Aurora kinases in general, or specifically Aurora A or B as a single agent. In order to optimize the efficacy and selectivity of oxostephanine against cancer cells, a combinatorial approach could be employed based on what has been found for other Aurora kinase inhibitors. For example, the inhibition of Aurora A in combination with the PI3Kα inhibitor BYL719, enhances apoptosis in breast cancer models [44]. Taken together, our data suggests that oxostephanine (**1**) provides a lead structure that could be optimized as a novel therapeutic in breast cancer and potentially other cancers.

### 2.6. In Vitro Anti-Plasmodial Activity of Isolated Compounds from S. dielsiana Y.C. Wu

Both MB2L-CH and MB2L-B fractions showed similar activity with IC_50_ values in the range of 4.5 to 7.9 μg/mL and 5.8 to 7.1 μg/mL against chloroquine-sensitive 3D7 and chloroquine-resistant W2 strain, respectively (Table 1). Of the seven alkaloid isolates, thailandine (**2**), crebanine (**4**), *O*-methylbulbocapine (**5**), and palmatine chloride (**6**) show strong activity against both 3D7 and W2 (Table 3). Thailandine (**2**) is the most active alkaloid on both the 3D7 and W2 *P. falciparum* strains with IC_50_ of 0.24 ± 0.04 µM and 0.22 ± 0.02 µM, respectively. Thailandine acted equally against chloroquine-susceptible and chloroquine-resistant *P. falciparum* parasites, suggesting that its antimalarial activity was independent on the mechanism of resistance to chloroquine. In contrast, stephanine (**3**) is not effective on chloroquine-resistant W2 strain, albeit very effective on chloroquine-sensitive 3D7. Thailandine (**2**) showed a low selectivity index (SI) ranged from 12 to 23 between cells and parasites (Table 3). When compared to its cytotoxicity against human cells, thailandine’s SI against *P. falciparum* is in the range of 12.6-fold (3D7 vs. 184B5) to 22.8-fold (W2 vs. MCF10A) (Table 3), indicating that it may be relatively safe. Our data thus show clearly that thailandine (**2**) is more active against *P. falciparum* parasites than stephanine (**3**), although the strong anti-malarial activity of stephanine (**3**) was recognized previously [32,34,45]. Crebanine (**4**), *O*-methylbulbocapine (**5**) and palmatine chloride (**6**) also show decent anti-plasmodial activities against both 3D7 and W2 with a low cytotoxic activity against 184B5 and MCF10A cells and, thus, with good selectivity (especially selective index SI > 20 on 3D7 strain, Table 3). Therefore, these compounds can also be good leads for further development. Oxostephanine (**1**) and tretrahydraoplamatine (**7**) were not active against *P. falciparum* parasites with IC_50_ value in the range of 63.9 to 275.5 µM against 3D7 and 215.5 to 226 µM against W2, respectively (Table 3).

Human Aurora kinase inhibitors, including hesparadin and its analogs, have already displayed excellent potency (from 0.01 to 1.6 µM µM) against *P. falciparum* parasites (chloroquine susceptible D6 strain) [8]. Oxostephanine (**1**), which shows the best anti-proliferative activity against cancer cells (HeLa, MCF7, MDA-MB-231, and MDA-MB-468) and which inhibits Aurora kinase activity, demonstrated poor activity against 3D7 and W2 strains. The modes of action between anti-proliferative and anti-plasmodial activities seem to be different. Thailandine (**2**), which shows the best anti-plasmodial activity, also displays significant cytotoxicity against cancer cells. The target of thailandine is still unknown in *P. falciparum*. But, the lysosomotropic properties of thailandine, due to quaternary nitrogen, could be involved in anti-plasmodial activity. Thailandine could inhibit endocytosis in parasites, like chloroquine, mefloquine, or artemisinin [46].

### 2.7. Thailandine as A Potential Anticancer Agent

Thailandine (**2**) shows significant cytotoxicity against cancer and non-cancer cell lines with IC_50_ values ranging from 0.78 to 7.11 μM (Table 2A). This compound is the most active compound on both *P. falciparum* strains with IC_50_ of 0.22 µM against 3D7 and 0.24 µM against W2 (Table 3). However, we were unable to isolate enough thailandine (**2**) from the MB2L-B fraction to pursue mechanistic studies. Thailandine remains of interest as both an anti-malarial and anti-cancer agent. Since thailandine (**2**) is electron-deficient and charged, it is possible that the positive charge of the molecule makes it more effective against certain sub-types of cancer [39]. Based on our work and others [31], thailandine may have an additional target that is specific to cells that have high expression of EGFR and/or are dependent on endocytic signaling. Since endocytic signaling depends on maintaining different pH within the endosome and lysosome, this pathway is sensitive to drugs that can act as weak bases and altering proton concentration. The quaternary nitrogen of thailandine (**2**) may be lysosomotropic, like ammonium chloride and other weak bases, and prevent lysosomal and endocytic signaling [47]. Thailandine’s cell killing action could be through the inhibition of EGFR signaling through the inhibition of endocytosis. Intriguingly, this may mean that thailandine can be utilized to target uniquely sensitive cancer cells (like MDA-MB468 and A549) using the high level of EGFR as a biomarker [31].

## 3. Material and Methods

### 3.1. General Experimental Procedures

The column chromatography (CC) was performed on silica gel Merck 60 (0.040–0.063 µm) and Sigma-Aldrich Sephadex LH-20. All thin layer chromatography (TLC) analyses were carried out on DC-AlufolienKieselgel 60 F254 (Merck, New Jersey, USA) and detected with a UV at 254 and 365 nm, followed by spraying with Dragendorff’s reagent for alkaloid detection. The ^1^H- and ^13^C-NMR spectra of isolated compounds in CDCl_3_ and/or CD_3_OD, DMSO-*d*_6_ solvent were recorded on Bruker AM 500 MHz spectrometer (Bruker, Fällanden, Switzerland) using TMS as the internal reference. Accurate, high resolution, and MS/MS mass measurements were carried out with an LTQ-Orbitrap mass spectrometer (Thermo Fisher Scientific, Inc., Bremen, Germany).

### 3.2. Plant Material

The leaves of *S. dielsiana* Y.C. Wu were collected in Ba Vi, Hanoi, Vietnam in March, 2015 and the identity was confirmed by Dr. Nguyen Quoc Huy, Hanoi University of Pharmacy. A voucher specimen, Nr. SD01/2015 is deposited in Institute of Chemistry, Vietnam Academy of Science and Technology, Vietnam.

### 3.3. Extraction and Isolation

The extraction and isolation of alkaloid compounds from the methanol extracts of *S. dielsiana* leaves were conducted as described previously [34,38]. Briefly, the dried and powdered leaves (3.0 kg) of *S. dielsiana* Y.C. Wu were macerated with methanol/water (85:15 *v:v*), at room temperature, three times (5 L, 24 h each time). The combined methanol extracts were evaporated under reduced pressure at 50 °C, and the methanolic extract (MB2L) was further fractioned with solvents of increasing polarity: *n*-hexane, dichloromethane, and *n*-butanol, successively. The organic solution was then concentrated under vacuum to give MB2L-H, MB2L-CH, and MB2L-B fractions. All these fractions were evaluated for their anti-proliferative activity. The MB2L-CH and MB2L-B fractions, the most effective fractions (Table 1), were further purified by repeated chromatography on silica gel columns to get the pure compounds [38]. The compounds **1**, **2**, and **6** and compounds **3**, **4**, **5**, and **7** were isolated from the MB2L-B and MB2L-CH fraction, successively. The chemical structures of these seven alkaloids, such as oxostephanine (**1**), thailandine (**2**), stephanine (**3**), crebanine (**4**), *O*-methylbulbocapnine (**5**), palmatine (**6**), and tetrahydropalmatine (**7**) were elucidated and confirmed by spectroscopic methods, including MS, ^1^H and ^13^C-NMR and compared to the reported references [34,38] and standard compounds (Supplementary Tables S1 and S2). The structures of isolated compounds **1**–**7** are illustrated in Figure 1.

### 3.4. Cell Lines and Cell Culture

In vitro anticancer study: The in vitro anti-proliferative tests were carried out as described previously [15], using aliquots of the cell lines used therein. Briefly, HeLa, MDA-MB-231 and MDA-MB-468, MCF7, and 184B5 and MCF 10A cell lines were purchased from ATCC. The identity of the cell lines presented in this study were verified by short tandem repeat profiling by Genetica DNA Laboratories (www.celllineauthentication.com) (April 8, 2015). MCF10A was grown in DMEM F12 medium supplemented with 0.02 μg/mL epidermal growth factor, 10% FBS, 10 μg/mL insulin and 0.5 g/mL hydrocortisone. MDA-MB-468 were grown in DMEM supplemented with 10% FBS and antibiotics cocktail (100 μg/mL streptomycin, 100 units/mL penicillin and 250 ng/mL amphotericin B). SRB cell proliferation assays were carried out as previously described [34,48,49], with some minor modifications. The concentration range for all tested cell lines were from 100–0.39 μg/mL for extracts and 100–0.39 μM for pure compounds. Paclitaxel (Cayman Chemical, Ann Arbor, MI, USA) (range: 100–0.39 nM) and chloroquine (Sigma Aldrich, Oakville, ON, Canada) (range: 100–1.56 μM) were used as positive controls. All experiments were carried out at least twice.

In vitro anti-plasmodial study: The two *P. falciparum* strains, the chloroquine-susceptible 3D7 (isolated in West Africa; obtained from MR4, Manassas, VA, USA), and the chloroquine-resistant strain W2 (isolated in Indochina; obtained from MR4, Manassas, VA, USA), were maintained in culture in RPMI 1640 (Invitrogen, Paisley, UK), supplemented with 10% human serum (Abcys S.A. Paris, France) and buffered with 25 mM HEPES and 25 mM NaHCO_3_. Parasites were grown in A-positive human blood (Etablissement Français du Sang, Marseille, France) under controlled atmospheric conditions that consisted of 10% O_2_, 5% CO_2_, and 85% N_2_ at 37 °C with a humidity of 95%.

Cell cycle analysis: Propidium iodide staining and flow cytometry analysis were carried out as described previously [34,48] with minor modifications. Briefly, 250,000 HeLa cells (50,000 cells/mL) were treated with 10 μM oxostephanine (**1**) and harvested at the indicated time points. All experiments were carried out at least twice.

Western Blot analysis: Western blotting was carried out as described previously [48]. The primary antibodies used were from Cell Signaling (Histone H3; p-Histone H3^S10^ and Phospho-Aurora A (Thr288)/Aurora B (Thr232)/Aurora C (Thr198)) and used at recommended dilution. For cell synchronization at prometaphase, HeLa cells were treated with 50 ng/mL nocodazole (NZ) (Santa Cruz Biotechnology) for 12 h. All experiments were carried out at least twice.

Annexin V staining: Cell death mechanism and cell proliferation assays were analysed using the IncuCyte Apoptosis Assay method using the Annexin V Green reagent (Essen Biosciences, Ann Arbor, MI, USA) and the IncuCyte S3 and IncuCyte ZOOM software (Version 2018B, Bohemia, NY, USA) according to supplier’s protocol. Statistical analysis was carried out in Graph Pad Prism 5. All experiments were carried out at least twice.

Molecular modeling: Docking was carried out using PyRx (0.9.8), utilizing Autodock 4.2 and the default PyRx settings [50]. All small molecules were energy minimized before docking. The docking box was drawn around the whole protein to allow for blind docking. Docking representations were exported as PDB files and displayed in 2D using LigPlot+ [51].

In vitro kinase assay: The Aurora A and B kinase assays were carried out using the Promega ADP-Glo based Kinase Activity Profiling system. The protocols for the Aurora A/(Myelin Basic Protein (MBP) and Aurora B/MBP assay kits were according to those recommended by the suppliers. The concentration range used was from 20 μM–39.1 nM in 384-well plates. The luminescent output from the kinase assay was read on a Biotek Synergy H4 plate reader.

In vitro anti-plasmodial assay: The two *P. falciparum* strains 3D7 and W2 were synchronized twice with sorbitol before use [52]. The clonality of these strains was verified both in our laboratory and in an independent laboratory from the Worldwide Anti-malarial Resistance Network (WWARN) by PCR genotyping of the polymorphic genetic markers msp1 and msp2 and microsatellite loci [53,54]. The compounds were re-suspended in methanol or DMSO 5% (*v/v*) and then diluted in methanol to obtain final concentrations ranging from 0.008 to 500 μg/mL (final concentration of 0.5% of methanol or DMSO). Chloroquine, mefloquine, and dihydroartemisinin were prepared and distributed into 96-well plates as described previously [55] and used for *P. falciparum* activity comparison. For the in vitro microtests, 200 μL of parasitized red blood cells (final parasitemia, 0.1%; final hematocrit, 1.5%) were aliquoted into 96-well plates pre-dosed with anti-malarial drugs (chloroquine, mefloquine and dihydroartemisinin) or natural compounds from *S. dielsiana*. The plates were incubated for 72 h at 37 °C in controlled atmospheric conditions 5% CO_2_, 10% O_2_, and 75% N_2_. After thawing the plates, haemolysed cultures were homogenized by vortexing the plates. Both the success of the drug susceptibility assay and the appropriate volume of haemolysed culture to use for each assay were determined for each clinical isolate during a preliminary HRP2 ELISA. Both the pre-test and subsequent ELISA assays were performed using a commercial kit (Malaria Ag Celisa, ref KM2159, Cellabs PTY LTD, Brookvale, Australia) according to the manufacturer’s recommendation. The optical density (OD) of each sample was measured with a spectrophotometer (Safire 2, Tecan, Lyon, France). The concentration at which the drugs were able to inhibit 50% of parasite growth (IC_50_) was calculated with the inhibitory sigmoid Emax model, with estimation of the IC_50_ through non-linear regression using a standard function of the R software (IC Estimator version 1.2). IC_50_ values were validated only if the OD ratio (OD at concentration 0/OD at concentration max) was greater than 1.6 and the confidence interval ratio (upper 95% confidence interval of the IC_50_ estimation/lower 95% confidence interval of the IC_50_ estimation) was less than 2.0. The anti-plasmodial in vitro assay was performed five times for each product. The IC_50_ values represented the mean value calculated from five experiments. According to Hout [56], a selectivity index (SI) was based on the ratio between anti-parasitic and cytotoxic activities. In the case of our study, a SI was calculated between non-tumorigenic cell line 184B5 and parasites 3D7 or W2 and/or MCF10A and these two parasites.

## 4. Conclusions

In conclusion, this is the first report on the cytotoxicity and anti-plasmodial activities of the methanol extract of *S. dielsiana* Y.C. Wu leaves, its fractions, and isolated compounds. The most active compounds isolated from this extract are two aporphine alkaloids, oxostephanine (**1**) and thailandine (**2**). Oxostephanine proves to be the most effective against most cancer cells, although its selectivity for cancer cells is low. Thailandine is effective only on sub-types of cancer cells including MDA-MB-468 and MCF7. However, it shows a good selectivity (SI > 12). The cytotoxic effect of oxostephanine is likely due to its inhibition of Aurora kinases activity, probably through its binding to the ATP-binding pocket. As for anti-plasmodial activity, thailandine is the most desirable among the seven isolates as it is effective on both chloroquine-susceptible 3D7 and chloroquine-resistant W2 *P. falciparum* strains. Furthermore, substantially high doses of thailandine are required to kill human cells, suggesting that it could be relatively safe. Together, our data suggest that oxostephanine and thailandine can be excellent lead compounds towards developing effective and safe anticancer and anti-malarial drugs. Further studies are needed to investigate the mode of action of thailandine on the human cancer cells as well as on *P. falciparum* parasites and its potential activity on hepatic stages and gametocytes.

## Figures and Tables

**Table 1:** Anti-proliferative activity of *S. dielsiana* Y.C. Wu extracts against cancer, non-cancer, and parasite cells.

Extracts	IC_50_ (μg/mL)							
	HeLa	MDA-MB231	MDA-MB-468	MCF7	184B5	MCF 10A	3D7	W2
MB2L	6.1 ± 1.2	7.8 ± 1.3	3.8 ± 0.5	5.9 ± 0.4	4.3 ± 1.8	8.9 ± 0.9	NT	NT
MB2L-H	>50	>50	>50	>50	>50	>50	NT	NT
MB2L-CH	1.1 ± 0.3	1.1 ± 0.6	0.6 ± 0.3	1.9 ± 0.6	2.2 ± 1.2	0.8 ± 0.6	4.5 ± 0.9	5.8 ± 0.4
MB2L-B	7.5 ± 0.6	9.4 ± 1.2	1.8 ± 1.0	14.4 ± 5.9	5.8 ± 0.6	12.5 ± 2.4	7.9 ± 1.3	7.1 ± 0.9

NT denotes not tested.

**Table:** **A**

Compounds	IC_50_ (μM ± SD)					
	HeLa	MDA-MB-231	MDA-MB-468	MCF7	184B5	MCF 10A
Oxostephanine **1**	1.76 ± 0.20	2.67 ± 0.29	2.26 ± 0.54	4.35 ± 1.20	1.66 ± 0.56	2.49 ± 0.11
Thailandine **2**	4.10 ± 0.40	7.11 ± 0.07	0.78 ± 0.12	1.99 ± 1.36	3.02 ± 0.10	5.01 ± 0.15
Stephanine **3**	3.33 ± 0.23	5.66 ± 0.16	7.14 ± 2.11	6.49 ± 0.43	6.25 ± 0.14	7.19 ± 0.33
Crebanine **4**	48.13 ± 2.38	38.94 ± 7.10	17.82 ± 4.63	30.50 ± 4.89	47.44 ± 2.83	47.16 ± 4.37
*O*-methylbulbocapine **5**	70.37 ± 11.40	56.59 ± 9.08	48.13 ± 1.69	39.36 ± 6.20	73.26 ± 0.47	59.47 ± 1.90
Palmatine chloride **6**	>100	>50	>50	>50	>50	>50
Tetrahydropalmatine **7**	>50	>50	>50	>50	>50	>50
Chloroquine	29.80 ± 0.70	28.90 ± 0.40	33.20 ± 0.40	>50	>50	40.50 ± 9.80
Paclitaxel ^a^	3.81 ± 0.42	1.58 ± 0.75	3.96 ± 0.32	2.71 ± 1.11	2.65 ± 1.91	1.67 ± 0.21

^a^ Paclitaxel with IC_50_ in nM. Chloroquine and paclitaxel are positive controls.

**Table:** **B**

	184B5				MCF10A			
Compounds	Hela	MDA-MB-231	MDA-MB-468	MCF7	HeLa	MDA-MB-231	MDA-MB-468	MCF7
Oxostephanine **1**	0.94	0.62	0.73	0.38	1.40	0.93	1.10	0.57
Thailandine **2**	0.73	0.42	3.90	1.50	1.20	0.70	6.40	2.50
Stephanine **3**	1.88	1.10	0.88	0.96	2.16	1.27	1.01	1.11
Crebanine **4**	0.99	1.22	2.66	1.56	0.98	1.21	2.65	1.55
*O*-Methylbulbocapine **5**	1.04	1.29	1.52	1.86	0.85	1.05	1.24	1.51
Chloroquine	<0.59	<0.57	<0.66	>1	1.40	1.40	1.20	<0.91
Paclitaxel	0.7	1.67	0.67	0.98	0.44	1.10	0.42	0.62

**Table 3:** IC_50_ values of the compounds isolated from MB2L-CH and MB2L-B against *Plasmodium falciparum* 3D7 and W2 strains, and 184B5 and MCF10A non-cancer cell lines.

Compounds	IC_50_ (μM ± SD)				SI			
	3D7	W2	184B5	MCF 10A	3D7		W2	
					184B5	MCF10A	184B5	MCF10A
Oxostephanine **1**	63.91 ± 18.38	215.54 ± 31.49	1.66 ± 0.56	2.49 ± 0.11	0.026	0.039	0.007	0.016
Thailandine **2**	0.24 ± 0.04	0.22 ± 0.02	3.02 ± 0.10	5.01 ± 0.15	12.58	20.86	13.73	22.77
Stephanine **3**	0.69 ± 0.15	1.32 ± 0.38	6.25 ± 0.14	7.19 ± 0.33	9.06	10.42	4.73	5.45
Crebanine **4**	1.56 ± 0.22	2.16 ± 0.38	47.44 ± 2.83	47.16 ± 4.37	30.41	30.23	21.96	21.83
*O*-methylbulbocapine **5**	2.81 ± 0.40	5.71 ± 0.62	73.26 ± 0.47	59.47 ± 1.90	26.07	21.16	12.83	10.42
Palmatine chloride **6**	1.25 ± 0.28	3.19 ± 0.64	>50	>50	>416.6	>416.6	>15.67	>15.67
Tetrahydropalmatine **7**	275.73 ± 14.43	226.09 ± 19.84	>50	>50	>0.18	>0.18	>0.22	>0.22
Chloroquine ^a^	0.021 ± 0.005	0.38 ± 0.03	>50	40.50 ± 9.80	>2380	1928	>131.6	106.50
Mefloquine ^a^	0.052 ± 0.009	0.022 ± 0.006	10.80 ± 0.75	NT	207.6	NA	490.9	NA
Dihydroartemisinin ^a^	0.002 ± 0.001	0.002 ± 0.001	NT	NT	NA	NA	NA	NA

SI denotes selectivity index between non-cancer cell line 184B5 (and MCF10A) parasites 3D7 or W2. NT denotes not tested. NA denotes not application. ^a^ Chloroquine, mefloquine, dihydroartemisinin are included as positive controls.

## Supplementary Materials

^1^H-NMR data (Table S1) and ^13^C-NMR data (Table S2) of isolated compounds from *S. dialsiana* Y.C. Wu are available online.
