# Supplementary material for: Cytotoxic and Anti-Plasmodial Activities of Stephania dielsiana Y.C. Wu Extracts and the Isolated Compounds
Source: Molecules. 2020 Aug 18;25(16):3755. doi: 10.3390/molecules25163755 (PMC7465040; doi:10.3390/molecules25163755)
Supplement: Supplementary file 1 [file molecules-25-03755-s001.pdf]

Supplementary Material

Cytotoxic and Anti-plasmodial Activities of *Stephania dielsiana* Y.C. Wu extracts and the Isolated Compounds

James Knockleby[1,2], Bruno Pradines[3,4,5,6], Mathieu Gendrot[3,4,5], Joel Mosnier[3,4,5,6], Thanh Tam Nguyen[7], Thi Thuy Trinh[7], Hoyun Lee[1,8*] and Phuong Mai Le[9*]

[1] Health Science North Research Institute, 56 Walford Road, Sudbury, ON P3E 2H2, Canada; jknockleby@hsnri.ca

[2] Department of Chemistry and Biochemistry, Laurentian University, 935 Ramsey Lake Road, Sudbury, ON P3E 2C6, Canada

[3] Département Microbiologie et Maladies Infectieuses, Institut de Recherche Biomédicale des Armées, 13005 Marseille, France; bruno.pradines@gmail.com (B.P.); ma.gendrot@laposte.net (M.G.); joelmosnier@orange.fr (J.M.)

[4] Aix Marseille University, IRD, SSA, AP-HM, VITROME, 13005 Marseille, France

[5] IHU Méditerranée Infection, 13005 Marseille, France

[6] Centre National de Référence du Paludisme, Institut de Recherche Biomédicale des Armées, 13005 Marseille, France

[7] Graduate University of Science and Technology and Institute of Chemistry, Vietnam Academy of Science and Technology, 18 Hoang Quoc Viet Road, 70750 Hanoi, Vietnam; nttam76@yahoo.com (T.T.N.); thuy@ich.vast.vn (T.T.T.)

[8] Departments of Medicine, University of Ottawa Medical School, Ottawa, ON K1H 5M8, Canada

[9] National Research Council Canada, 100 Sussex Drive, Ottawa, ON K1A 0R6, Canada

* Correspondence: hlee@hsnri.ca (H.L.); PhuongMai.Le@nrc-cnrc.gc.ca (P.M.L); Tel.: 1 705 523 7300 (H.L.); 1 613 991 9406 (P.M.L.)

Received: 30 July 2020; Accepted: 13 August 2020; Published: date

List of Tables: Table S1: ^{1}H-NMR data of the isolated compounds **1 – 7** from *Stephania dielsiana* (500 MHz, δ, ppm, *J*/Hz)

Table S2: ^{13}C-NMR data of the isolated compounds **1 – 7** from *Stephania dielsiana* (125 MHz, δ, ppm)

Table S1. ^{1}H-NMR data of the isolated compounds **1 – 7** from *Stephania dielsiana* (500 MHz, δ, ppm, *J*/Hz)

Position	**1**[a]	**2**[b]	**3**[c]	**4**[c]	**5**[b]	Position	**6**[a]	**7**[c]
	δ_H	δ_H	δ_H	δ_H	δ_H		δ_H	δ_H
1						1	7.45 (1H, s)	6.74 (1H, s)
3	7.46 (1H, s)	7.63 (1H, s)	6.55 (1H, s)	6.52 (1H, s)	6.65 (1H, s)	3		
3a						4	6.74 (1H, s)	6.62 (1H, s)
4	7.91 (1H, d, *J* = 5.0)	8.39 (1H, d, *J* = 6.5 Hz)	3.14 – 3.03 (m*) 2.62 (1H, dd, *J* = 16.0, 3.5)	3.16-3.03 (m*) 2.62 (1H, dd, *J* = 16.0, 3.5)	3.35-3.28 (1H, m) 2.98 (1H, dd, *J* = 17.5, 4.0)	4a		
5	8.73 (1H, d, *J* = 5.0)	8.73 (1H, d, *J* = 6.5 Hz)	3.14-3.03 (m*) 2.51 (1H, dd, *J* = 11.5, 3.5)	3.16-3.03 (m*) 2.52 (1H, dd, *J* = 11.5, 3.5)	3.81-3.78 (m*) 3.54 (1H, dt, *J* = 12.5, 4.5)	5	3.24 (3H, t, *J* = 6.5)	3.12 (1H, m) 2.62 (m*)
6a			3.14-3.03 (m*)	3.16-3.03 (m*)	4.33-4.31 (1H, m)	6	5.02 (1H, *J* = 6.5)	3.20(1H, m) 2.62 (m*)
6b						8	9.88 (1H, s)	4.24 (1H, d, *J* = 15.5) 3.52 (1H, d, *J* = 15.5)
7			3.68 (1H, dd, *J* = 15.0, 4.5) 2.20 (1H, dd, *J* = 14.5, 14.0)	3.67 (1H, dd, *J* = 14.5, 4.5) 2.29 (1H, dd, *J* = 14.5, 14.0)	3.81-3.78 (m*) 2.98 (1H, dd, *J* = 14.5, 14.0)	8a		
8					7.83 (1H, d, *J* = 8.5)	10		

9	7.26 (1H, J = 8.5 Hz)	7.34 (1H, d, J = 8.5 Hz)	6.83 (1H, d, J = 8.0)		6.99 (1H, J = 8.5)	11	7.71 (1H, d, J = 9.0)	6.79 (1H, dd, J = 8.5)
10	7.75 (1H, t, J = 8.0)	7.82 (1H, t, J = 8.0 Hz)	7.26 (1H, dd, J = 8.0, 8.0)	6.87 (1H, d, J = 8.5)		12	7.91 (1H, d, J = 9.0)	6.88 (1H, dd, J = 8.5)
11	8.27 (1H, d, J = 8.0)	8.30 (1H, d, J = 8.0 Hz)	7.71 (1H, d, J = 8.0)	7.80 (1H, d, J = 8.5)		12a		
11a						13	8.55 (1H, s)	3.36 (1H, dd, J = 16.0, 4.0)<br>2.83 (1H, dd, J = 15.5, 11.5)
11b						13a		3.52 (1H, m)
12	6.44 (2H, s)	6.66 (2H, s)	6.08 (1H, d, J = 1.5)<br>5.90 (1H, d, J = 1.5)	6.05 (1H, d, J = 1.5)<br>5.90 (1H, d, J = 1.5)	6.11 (1H, J = 1.0)<br>5.97 (1H, J = 1.0)	13b		
NCH_3		4.72 (3H, s)	2.57 (3H, s)	2.58 (3H, s)	3.18 (3H, s)	2-OCH_3	4.05 (3H, s)	3.89 (3H, s)
8-OCH_3	3.96 (3H, s)	4.05 (3H, s)	3.85 (3H, s)	3.82 (3H, s)		3-OCH_3	3.96 (3H, s)	3.86 (3H, s)
9-OCH_3				3.93 (3H, s)		9-OCH_3	4.18 (3H, s)	3.87 (3H, s)
10-OCH_3					3.88 (3H, s)	10-OCH_3	3.99 (3H, s)	3.85 (3H, s)
11-OCH_3					3.86 (3H, s)			

[a] Measured in DMSO-d_6. [b] Measured in CD_3OD. [c] Measured in $CDCl_3$. * Overlapped signals.

Table S2.^{13}C-NMR data of the isolated compounds **1 – 7** from *Stephania dielsiana* (125 MHz, δ, ppm)

Positions	**1**[a]	**2**[b]	**3**[c]	**4**[c]	**5**[b]	Positions	**6**[a]	**7**[c]
	δ_C	δ_C	δ_C	δ_C	δ_C		δ_C	δ_C
1	151.22	159.10	142.72	142.12	144.61	1	108.34	108.63
2	147.37	153.13	146.65	146.65	150.01	2	149.43	147.56
3	102.58	104.20	107.55	106.83	107.77	3	152.09	147.47
3a	134.47	141.79	126.45	126.43	120.69	4	110.67	111.38
4	122.89	126.71	29.16	29.02	26.90	4a	128.00	126.65
5	143.76	142.07	53.66	53.60	53.84	5	27.09	28.91
6a	145.79	142.59	61.84	61.89	62.99	6	56.23	51.42
6b	121.06	124.54	127.17	126.43	124.99	8	145.62	53.87
7	179.69	179.23	25.96	26.84	25.41	8a	121.87	127.56
7a	120.27	120.96	123.79	129.68	126.76	9	144.46	145.07
8	160.87	162.83	156.25	152.07	124.73	10	150.23	150.28
9	112.77	114.43	109.69	145.90	112.84	11	126.11	111.05
10	134.18	137.75	127.06	110.34	153.88	12	123.46	123.82
11	118.85	120.47	119.47	123.12	147.13	12a	133.48	128.35
11a	134.27	134.39	132.16	124.61	124.51	13	120.01	36.12
11b	106.76	109.69	116.58	116.54	117.59	13a	137.85	59.26
12	102.38	106.69	100.64	100.64	102.77	13b	118.77	129.44
NCH_3	-	50.04	43.96	43.82	42.22	2-OCH_3	56.75	56.07
8-OCH_3	55.87	56.89	55.59	60.72	-	3-OCH_3	56.23	55.86
9-OCH_3	-		-	55.78	-	9-OCH_3	62.32	60.15
10-OCH_3	-		-	-	56.40	10-OCH_3	56.88	55.82
11-OCH_3	-		-	-	61.19			

[a] Measured in DMSO-d_6. [b] Measured in CD_3OD. [c] Measured in $CDCl_3$.
